# Multicolor multifocal 3D microscopy using in-situ optimization of a spatial light modulator

**DOI:** 10.1038/s41598-022-20664-z

**Published:** 2022-09-29

**Authors:** M. Junaid Amin, Tian Zhao, Haw Yang, Joshua W. Shaevitz

**Affiliations:** 1 Department of Chemistry, Princeton University, Princeton, NJ 08544 USA; 2 Department of Physics, Princeton University, Princeton, NJ 08544 USA; 3 Lewis-Sigler Institute for Integrative Genomics, Princeton University, Princeton, NJ 08544 USA

**Keywords:** Lasers, LEDs and light sources, Optical techniques, Imaging and sensing, Microscopy

## Abstract

Multifocal microscopy enables high-speed three-dimensional (3D) volume imaging by using a multifocal grating in the emission path. This grating is typically designed to afford a uniform illumination of multifocal subimages for a single emission wavelength. Using the same grating for multicolor imaging results in non-uniform subimage intensities in emission wavelengths for which the grating is not designed. This has restricted multifocal microscopy applications for samples having multicolored fluorophores. In this paper, we present a multicolor multifocal microscope implementation which uses a Spatial Light Modulator (SLM) as a single multifocal grating to realize near-uniform multifocal subimage intensities across multiple wavelength emission bands. Using real-time control of an in-situ-optimized SLM implemented as a multifocal grating, we demonstrate multicolor multifocal 3D imaging over three emission bands by imaging multicolored particles as well as *Escherichia coli* (*E. coli*) interacting with human liver cancer cells, at $\sim 2.5$ multicolor 3D volumes per second acquisition speed. Our multicolor multifocal method is adaptable across SLM hardware, emission wavelength band locations and number of emission bands, making it particularly suited for researchers investigating fast processes occurring across a volume where multiple species are involved.

## Introduction

Multifocal microscopy allows simultaneous 3D volume imaging at a diffraction limited resolution^1–4^. This widefield imaging modality typically uses a diffraction grating, known as a multifocal grating, in the emission path which splits the emission light into multiple diffraction orders that are imaged as subimages side-by-side onto a camera sensor. These subimages correspond to unique object planes which are separated in object space by a distance $\Delta z$. This method allows volume imaging speeds reaching hundreds of frames per second, limited only by the camera, which makes this technique attractive for many researchers.

**Figure 1:**
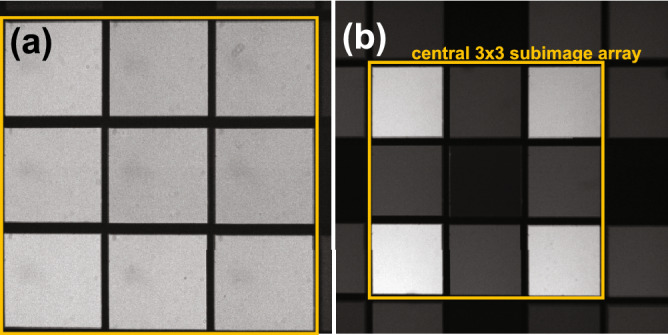
Example images showing the effect deploying an SLM-displayed multifocal grating pattern for an emission filter for which the pattern is not designed for. Here, an SLM pattern is displayed which was optimized for a $3\times 3$ subimage array using an emission filter having $\lambda _\text {c}=671$ nm and SLM calibration settings giving $0-2\pi$ relative phase control for $\lambda = 696$ nm. Using this pattern, corresponding bright-field mode multifocal images are obtained using emission filters having (**a**) $\lambda _c=671$ nm, which is the pattern design wavelength giving $M=0.81$, and (**b**) $\lambda _c=512$ nm, which is far from the design wavelength giving $M=0.07$. The orange rectangle indicates the camera region occupied by the central $3\times 3$ subimage array.

Although single emission wavelength multifocal imaging has been demonstrated^1,2^, extending multifocal microscopy to multicolor imaging applications has been challenging thus far due to the characteristic nature of the multifocal grating. The diffractive pattern on this grating is typically designed to provide uniform subimage intensities for a single emission wavelength ($\lambda$) band having a central emission wavelength $\lambda _\text {c}$. When these gratings are used for imaging samples having multicolored species, they lead to non-uniform subimage intensities in wavelengths for which the grating is not designed^5,6^. An example of this issue is shown in Fig. 1 for an SLM-based multifocal microscope optimized for $\lambda _\text {c}=671$ nm, where a metric *M* is used to quantify the intensity uniformity (see "Multicolor multifocal microscope" section for metric description).

One approach to dealing with this issue is to use the same grating pattern for imaging via multiple emission bands and implement post-processing intensity scaling in the resulting non-uniformly illuminated subimages obtained in the “off-design“ emission band^5,6^. Low-intensity subimages lead to poor signal-to-noise performances and loss of information, however. Another approach involves designing a pattern for custom-fabricated gratings to afford uniform subimage intensities in two separate predetermined emission channels^7^. A third approach involves splitting the emission into different color channels and using individual multifocal gratings for each color path. This approach allows precise multifocal multicolor imaging which is demonstrated for neuronal imaging^8^. Encouraged by these prior-art advances, we envision a new-generation multicolor multifocal platform that uses a single multifocal grating to allow uniform subimage intensities at any emission wavelength band of choice as well as any number of emission wavelengths.

In this paper, we present such a multicolor multifocal microscope platform concept which allows uniform subimage intensities across multiple tunable emission bands using the same grating optics. This is accomplished by using in-situ optimization of an SLM deployed as the multifocal grating^4^ and generalizing the framework for an in-situ multicolor implementation. In principle, our approach is adaptable to any $\lambda _\text {c}$ and number of emission channels. These characteristics make this multicolor multifocal microscope a useful tool for various imaging applications involving multicolor species. In the rest of the paper, we first describe the optical setup, followed by a description of single-color multifocal microscopy. We then describe our approach towards multicolor multifocal operations, before presenting the experimental demonstrations. This is followed by a discussion section before a brief conclusion paragraph which summarizes the work.

## Multicolor multifocal microscope

### Optical diagram

**Figure 2:**
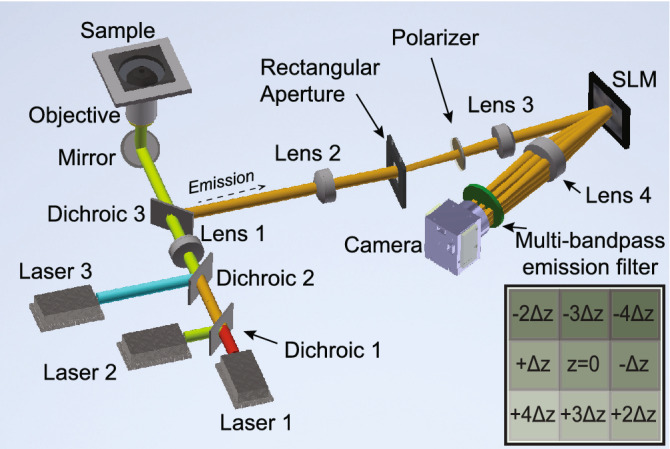
Optical diagram of the multicolor multifocal microscope. Inset shows the conjugate object plane arrangement for a $3\times 3$ subimage array imaged on a camera.

The multicolor multifocal microscope design is shown in Fig. 2. Excitation light from multiple laser sources (three lasers are shown in Fig. 2) are directed through dichroic optics towards a sample via a microscope objective. Lens 1 focuses the excitation light onto the back focal plane of the objective to illuminate the full sample imaging field of view. Emission light from fluorophores in the sample is collected using the same objective and reflected off Dichroic 3 towards Lens 2 which forms an image at the Rectangular Aperture plane. This Rectangular Aperture limits the imaging field of view to prevent multifocal subimages from overlapping. The emission light is then directed onto the SLM placed at the Fourier plane via Lens 3. A linear polarizer is placed in the emission path to ensure phase-only operation of the SLM. A grayscale grating pattern displayed on the reflective SLM results in multiple diffraction orders of the emission light which are imaged onto a camera by Lens 4 through a multi-bandpass emission filter. The inset in Fig. 2 shows the camera placement of the subimages formed, relative to the $z = 0$ plane, which are conjugate to unique object planes separated by $\Delta z$. Optional brightfield/darkfield illumination (not shown in Fig. 2) can be incorporated in the optical design for additional multifocal modalities.

### Multifocal imaging over a single-wavelength emission band

Before presenting the multicolor multifocal pipeline, we first describe conventional single-wavelength emission band multifocal imaging. To begin, using the in-situ iterative algorithm^4^, an optimized SLM multifocal grating pattern specific to the emission band is acquired. Briefly, the SLM is calibrated using the calibration settings specific to the emission wavelength $\lambda _\text {c}$ to achieve 0–2$\pi$ relative phase control corresponding to 0–255 grayscale patterns. The optimized pattern is displayed on the SLM and can readily be edited to vary $\Delta z$ without affecting the subimage intensities^4^. Once a desired $\Delta z$ is chosen, the pattern is displayed on the SLM and is kept unchanged throughout an imaging experiment until it finishes or a different $\Delta z$ is desired.

### Multifocal imaging across multiple emission bands

By and large, there are two steps involved in generalizing multifocal imaging for arbitrary multiple emission bands using an SLM-based technology. One is the calibration of the input control voltage to the phase of each SLM pixel whereas the other is the generation of SLM-generated grating pattern for multifocal image projection. In our implementation, the former is accomplished through a separate control experiment and the latter is carried out *n situ*. To begin, optimized SLM patterns are obtained separately for each of the emission bands. For multicolor multifocal imaging, the same SLM calibration settings are used for operations across multiple emission bands. This is possible because of the adaptability the in-situ iterative algorithm^4^ which provides grating patterns giving near-uniform subimage intensities regardless of SLM calibration settings.

**Figure 3:**
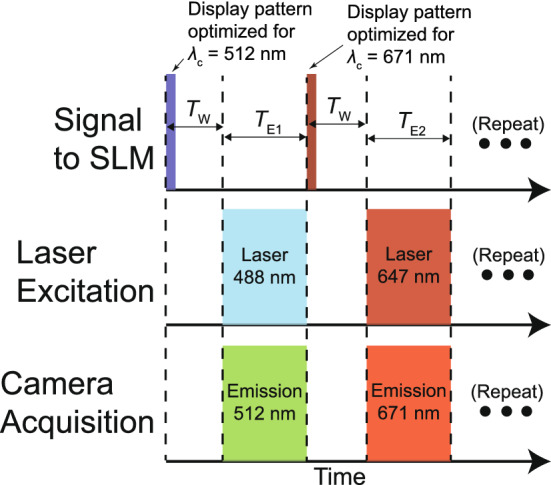
Example timing diagram for a two-color multifocal microscope.

Once optimized SLM patterns are obtained for the different emission bands using the same SLM calibration settings, these patterns are stored and synchronously displayed on the SLM with a multicolor excitation and image acquisition routine. A timing diagram of an example two-colored multifocal microscope illustrating this concept is shown in Fig. 3. In Fig. 3, the two-colored timing diagram assumes excitation lasers of wavelength 488 nm and 647 nm, while a multi-bandpass emission filter has emission bands located at $\lambda _\text {c}$ values of 512 nm and 671 nm. To begin the multicolor multifocal imaging routine, the SLM pattern optimized for $\lambda _\text {c}=512$ nm is first sent to the SLM for display. A wait period of time $T_\text {W}$ is subsequently needed to account for the $\sim$16.67 ms (60 Hz) addressing rate of typical SLMs as well as the response time of the liquid crystals in the SLM^9^. After $T_\text {W}$ has passed, the 488 nm laser is turned on while the camera acquires an image having an exposure time $T_\text {E1}$. Once the image corresponding to 488 nm excitation is acquired, the 671 nm centered emission band optimized grayscale is sent to the SLM. A wait time of $T_\text {W}$ passes before the 647 nm laser turns on and the camera acquires an image of exposure time $T_\text {E2}$ corresponding to the emission band centered at 671 nm. This cycle is repeated until the desired number of images are acquired. Note that this sequence can accommodate additional wavelength emission bands, each having a corresponding wait time $T_\text {W}$ as well as acquisition time. These features make this method universally adaptable for a large variety of imaging requirements. This concludes the multicolor multifocal pipeline.

### Multifocal image uniformity metric *M*

For a measured subimage intensities {$I_\text {m,i}$}, with $i=$ {1, 2, ... N} where *N* is the number of subimages, the metric *M* is used to quantify multifocal image intensity uniformity^4^:

1 $$\begin{aligned} M=\dfrac{\min \left( \{I_\text {m,i}\}\right) -I_\text {b}}{\max \left( \{I_\text {m,i}\}\right) -I_\text {b}} \end{aligned}$$

where $I_\text {b}$ is a measured background intensity. The higher the subimage intensity uniformity, the higher the *M*. $M=1$ indicates that all subimages have equal intensities.

## Results

The multicolor multifocal microscope is implemented on a home-built aluminum block as the microscope base and controlled using an in-house developed LabVIEW^10^ code. The SLM used is Holoeye Pluto-VIS-056 having a pixel size of 8 $\mu \hbox {m}$, which is calibrated using a manufacturer-provided file to give $2.3\pi$ relative phase control at 532 nm. This is meant to provide a linear $2\pi$ relative-phase control range for a wavelength of 611.8 nm using 8-bit grayscale patterns. We have empirically chosen this particular setting because it is near the mid-point of the emission band $\lambda _\text {c}$’s used in the experiments. These SLM calibration settings were kept fixed throughout the duration of the experiments, an important step of the multicolor multifocal design. The $\Delta z$ is set to 500 nm, and a grating period of $4\times 4$ SLM pixels is used. The remaining experimental components used in the setup are discussed in Methods. Furthermore, details describing how $\Delta z$ is controlled can be found in previous works^4,5^.

**Figure 4:**
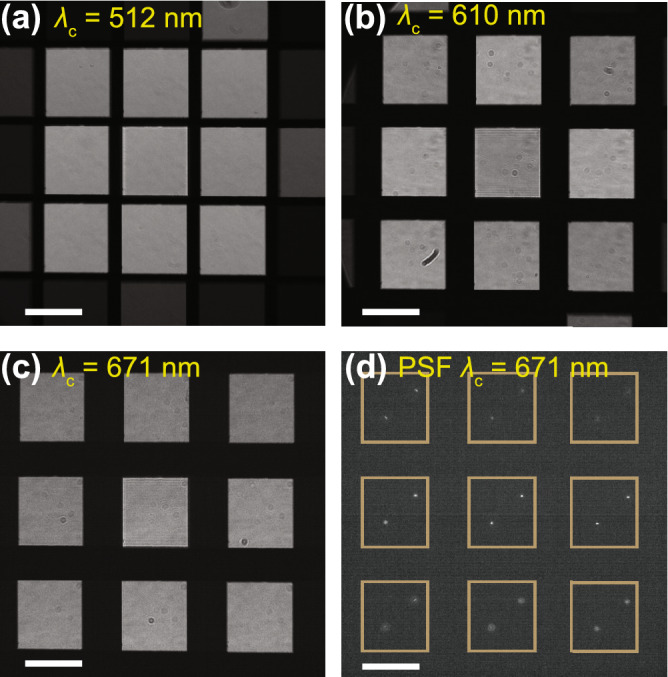
Using the same SLM calibration settings, shown are brightfield-mode multifocal images obtained using optimized patterns corresponding to (**a**) $\lambda _\text {c}=512$ nm with $M=0.91$, (**b**) $\lambda _\text {c}=610$ nm with $M=0.81$, (**c**) $\lambda _\text {c}=685$ nm with $M=0.79$, and (**d**) a multifocal image of a stationary 200 nm bead sample acquired at $\lambda _\text {c}$ =671 nm emission wavelength with $\Delta z$ = 900 nm indicating the PSF of the microscope with the beads in focus in the central subimage. The scale bar in each figure represents 20 $\mu \hbox {m}$.

Using brightfield illumination, optimal SLM patterns providing near-uniform intensities across multifocal subimages are obtained using the *in-situ* iterative algorithm^4,11^ for $\lambda _\text {c}$ values 512 nm, 610 nm and 671 nm. The corresponding brightfield-mode multifocal images are displayed in Fig. 4a, b, and c, exhibiting high * M* values and showing the near-uniform subimage intensities across the three empirically chosen emission bands. In principle, the method is applicable for any $\lambda _\text {c}$ location in the wavelength spectrum. Fig. 4d is a snapshot of a 200 nm fixed bead sample acquired using $\Delta z$ = 900 nm and $\lambda _\text {c}$ = 671 nm, indicating the lateral point spread function (PSF) of the system. Supplementary video 1 shows a collection of images acquired in a z-stack of the 200 nm fixed bead sampleshowing how the axial PSFs vary as the beads come in and out of focus in the subimages. We observe the PSF axial resolution across the 9 planes to be near-uniform (see Supplementary information). Note that the shape of the PSF is different across the subimages. This is due to the finite bandwidth of the emission filter which causes chromatic spreading arising from the grating pattern displayed on the SLM. The impact of emission filter bandwidth on the shape of the PSF in such systems is discussed thoroughly in previous work^12^.

The following experiments were performed following the Fig. 3 sequence, with $T_\text {W}$ set to 135  ms. This $T_\text {W}$ value was empirically found to afford high-performance multicolor imaging with our existing hardware, given the 16.67 ms addressing time and the >70 ms response time (10% to 90%) quoted in the SLM manual provided by the manufacturer.

**Figure 5:**
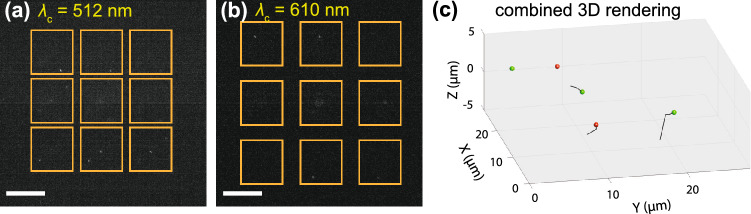
Example multifocal images from a sequence of two-color imaging of a mixture made up of yellow-green (505/515) and red (580/605) $\mu$m-sized fluorescent particles. (**a**) $\lambda _\text {c}=512$ nm channel, and (**b**) $\lambda _\text {c}=610$ nm, (**c**) 3D rendering of the combined multicolor multifocal images (**a**) and (**b**) showing the $\lambda _\text {c}=512$ nm channel particles in green and $\lambda _\text {c}=610$ nm in red. The black tail of each particle indicates the corresponding moving trajectory. The scale bar in each figure represents 20 $\mu \hbox {m}$.

Using the obtained optimized SLM patterns, we first demonstrate multicolor multifocal imaging of two-colored 100 nm diameter particles freely diffusing in solution. A diluted mixture of yellow-green (505/515) and red (580/605) $\mu$m-sized particles (FluoroSphere, ThermoFisher Scientific) is sandwiched between two coverslips (ThermoFisher), and imaged using the microscope (see Methods for detailed sample preparation). The different colored particles were imaged using the sequence shown in Fig. 3 and the SLM patterns optimized for 512 nm and 610 nm, which were illuminated by Laser 3 and Laser 2, respectively (cf. Fig. 2). Both $T_\text {E1}$ and $T_\text {E2}$ were set to 50 ms. A single cycle of multicolor 9-plane multifocal image took $\sim$400 ms, which includes acquisition of both color channels. Supplementary video 2 shows an image acquisition sequence of only the 512 nm channel at $3\times$ real-time speed, while the 605 nm channel image sequence from the same dataset is shown in Supplementary video 3. Individual frames from both channels are shown in Figs. 5a and  b. Supplementary video 4 shows the combined volume rendering, using in-house code written in MATLAB^13^, of the multicolored multi-particle dataset with the 512 nm channel shown as green and the 605 nm channel shown as red. A snapshot from the 3D rendered (Supplementary video 4) movie is shown in Fig. 5c. This 2.5 volumes per second multicolored particle imaging demonstration shows the utility of our system for potentially tracking multiple types of species moving over a large volume.

**Figure 6:**
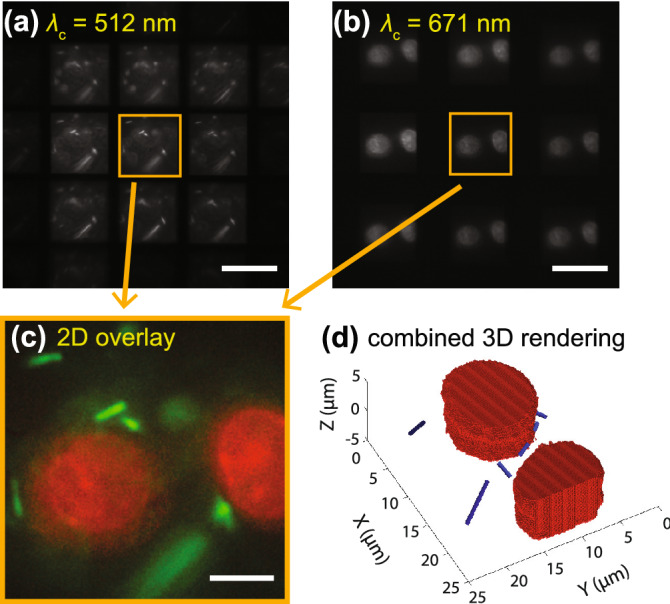
Example multifocal images from a sequence of two-color imaging of a sample consisting *E. coli* and human liver cells. (**a**) $\lambda _\text {c}=512$ nm channel, and (**b**) $\lambda _\text {c}=671$ nm, (**c**) overlay of the central subimages corresponding to $z=0$ plane from both (**a**) and (**b**). The *E. coli* is colored green while the human liver cells are colored red, (**d**) 3D rendering of the images in (**a**) and (**b**) showing the mammalian cells (red) and the *E. coli* cells (blue). The scale bars in (**a**) and (**b**) represent 20 $\mu$m, the scale bar in (**c**) represents 5 $\mu \hbox {m}$.

Next, we imaged a sample containing the bacteria *E. coli* and human liver cells (see Methods for sample preparation details) using the multicolor multifocal imaging platform. It has been reported that the engineered bacteria can deliver the drug to the trageted tumor to inhibit the growth of the tumor^14,15^. The proof-of-principle experiment in this section is expected to show the capability of our multicolor multifocal imaging platform that can unveil the underlying mechanism of such interactions between bacteria and mammalian cells. Fluorescent *E. coli* excited by Laser 3 were imaged in the 512 nm channel while human liver cells were excited by Laser 1 and imaged in the 671 nm emission channel. Both $T_\text {E1}$ and $T_\text {E2}$ were set to 100 ms for this demonstration. Supplementary video 5 shows the 9-plane multifocal images of *E. coli*, while Supplementary video 6 displays the acquisition of human liver cells from the same multicolor dataset. Selected frames from the multicolor data acquisition are shown in Fig. 6. We also overlayed the central subimages (corresponding to only the $z=0$ plane) from both channels to show the spatial relationship of the two species (Fig. 6c and Supplementary video 7), where the *E. coli* is colored green and the human liver cells are colored red. In particular, Supplementary video 7 shows the *E. coli* pushing and pulling on the human liver cells. Note that the remaining 8 planes from each channel were unused in Fig. 6c as part of the overlay process, showing the large volume of information available to visualize and observe such dynamics. Fig. 6d shows a snapshot of a 3D rendered movie (Supplementary video 8) computed using the combined multicolor multifocal data as input to an in-house MATLAB rendering code. The large red structures in Fig. 6d represent the human liver cells while the blue structures around them represent the *E. coli*. The ability to acquire 3D volumetric multicolor data such as shown in Fig. 6 makes this technique suitable for investigating inter-species dynamics across a variety of systems.

## Discussion

The multicolor multifocal imaging routine presented here overcomes prior-art issues of non-uniform subimage intensities during multicolor image acquisitions. Our in-situ optimization method for SLM as the multifocal grating, which allows high performance multicolor multifocal imaging, is adaptable across multiple microscope types and SLM hardware manufacturers, allowing access to many researchers. Our design allows the use of the same SLM calibration settings for different wavelengths of operations. Empirically, the exact choice of the $0-2\pi$ calibration wavelength is not important as long as the calibration wavelength for $2\pi$ relative phase control is near the desired emission $\lambda _\text {c}$ values.

An important aspect of the SLM-based multifocal microscope is the efficient detection of signal photons arising from the sample. The in-situ iterative method used here gives $\sim$75$\%$ diffraction efficiency, which is the normalized sum of the intensities in the non-zeroth order subimages^4^. The light efficiency of our method, in which we include the zeroth order intensities into our calculations, is $\sim$85$\%$, i.e., 85% of the emission light reflected by the SLM is contained in the 9 subimages captured by the camera. Note that this calculation does not include transmission through the bandpass filter as well as the lens forming the image on the camera. Assuming a $\sim$95$\%$ reflectivity of the SLM quoted by the manufacturer, we get roughly 80% of the emitted light striking the camera to form a multifocal image. Splitting into 9 subimages gives approximately $\sim$9$\%$ of the emitted light per subimage. Although this reduction in subimage brightness is not an issue for bright samples, this can impact imaging of weakly fluorescent samples. In the latter case, the exposure time of the camera and/or the sample illumination will have to be increased in case the multifocal images appear to have low a signal-to-noise ratio. The losses in the emission path include light leaking into higher diffraction orders which are not used/captured by the camera as well as the imperfect SLM reflectivity. As this technology evolves, we anticipate this efficiency to increase further as new optimization algorithms are developed.

The multicolor imaging speeds depend significantly on the hardware used. In our current setup, we used $T_\text {W}=135$ ms. This wait time was obtained experimentally by measuring how long it took for a new SLM grating pattern to stabilize on the camera by evaluating the resulting *M* values. Different SLMs have different response times which depends on a range of factors including the liquid crystal pixel characteristics as well as the drive voltage settings. Deploying SLMs with faster response times will increase the volume imaging speed of the proposed multicolor multifocal routine. Furthermore, note that the location of subimages in the multifocal images changes shown for different $\lambda _\text {c}$ values as expected according to the grating equation^16^.

Finally, the large volumes of sample information provided by the multicolor multifocal microscope as well as their variation across camera regions due to different emission wavelength bands indicate a need for specialized post-processing techniques. Although researchers have implemented various reconstruction methods for multifocal imaging volumes^17,18^, the extension to multicolor imaging presents unique computational challenges yet opportunities for future research, such as deciphering 3D shapes from multiple focal plane images of different shaped objects interacting with each other (e.g., Fig. 6). Note that in this manuscript, we extend the utility of the SLM-based multifocal microscope to realize multicolor multifocal imaging. In addition to having no objective or sample motion necessary for volumetric imaging, the advantages of having a spatial light modulator perform this task are numerous including customizable emission bands and focal spacing between the planes being imaged.

## Conclusion

In conclusion, an SLM-based multicolor multifocal microscopy platform is presented. Using *in-situ* optimization of an SLM used as the multifocal grating, we show that it is possible to provide near-uniform multifocal subimage intensities for three emission wavelength bands in the visible range using a single multifocal grating. Using this idea, as demonstrated by our platform, researchers can optimize multifocal experiments according to desired dye emission wavelengths as well as the number of emission channels. This promises to be a useful tool for researchers probing volume dynamics across a variety of biological and material systems.

## Methods

### Optical setup components

The components used in the experimental setup are: Laser 1: Cobolt 06-01 series with wavelength of 647 nm and maximum power of 130 mW, Laser 2: Cobolt 08-01 series with wavelength of 561 nm and maximum power of 100 mW, Laser 3: Cobolt 06-01 series with wavelength of 488 nm and maximum power of 120 mW, Dichroic 1: Semrock Di03-R561-t3-25x36, Dichroic 2: Semrock’s Di03-R488-t3-25x36, Dichroic 3: Semrock’s Di03-R405/488/561/635-t3-25x36, Microscope objective: Leica HC PL APO 100x magnification with Numerical Aperture adjustable between 0.7 to 1.4, focal length of Lens 1: 150 mm, focal length of Lens 2, Lens 3 and Lens 4: 200 mm, Rectangular Aperture from Ealing (Hyland Optical Technologies), Linear Polizer: model LPVISE100-A from Thorlabs, Camera: Orca Flash 4 V3 from Hamamatsu. Holoeye’s Pluto-VIS-056 was used as the SLM in this microscope, which had a pixel size of 8 $\mu m$. The Multi-bandpass filter deployed was custom-made by Alluxa having the central wavelengths ltocated at 460 nm, 512 nm, 610 nm and 671 nm, with corresponding bandwidths of 21 nm, 15 nm, 16 nm and 20 nm, respectively. The Rectangular Aperture was controlled such that the total field of view per subimage was set to $\sim$27$\mu m^2$.

### Multi-particle sample preparation

Yellow-green fluorescent (505/515) carboxylate-modified FluoSpheres of 0.1 $\mu$m and Red (580/605) carboxylate-modified FluoSpheres of 0.1 $\mu \hbox {m}$ were purchased from ThermoFisher Scientific. 10 $\mu \hbox {L}$ of 10$^4\times$ diluted yellow-green particles was mixed with 20 $\mu \hbox {L}$ of $10^4\times$ diluted red particles. 10 $\mu \hbox {L}$ of this mixture was then added to 90 $\mu \hbox {L}$ of Thiodiethanol (SigmaAldrich). 15 $\mu$L of this mixture is dropped on a 22 mm $\times$22 mm coverslip (ThermoFisher) using a pipette. A 18 mm $\times$ 18 mm coverslip was then placed on top of this droplet which was then spread throughout the top coverslip. The sample was imaged via the bottom coverslip.

### Bacteria/liver cell sample preparation

A pipette tip was used to scrape a stock of frozen *E. coli* MG1655 KanR having the Green Fluorescent Protein (GFP) plasmid pWR20. The pipette tip was then dipped into 3 mL of Lysogeny Broth (LB) media in a tube. 15 $\mu \hbox {L}$ of Kanamycin was added to this mixture to neutralize the non-GFP plasmid carrying bacteria and incubated at $37^{\circ }\hbox {C}$ for 18 hours. After the incubation period, 15 $\mu \hbox {L}$ of the bacteria solution was added to 50 $\mu \hbox {L}$ of the liver-cell solution (see description below). This volume was dropped on a coverslip which was then placed on the microscope stage for imaging.

HEP G2 human liver cancer cells (HB-8065) were purchased from ATCC and cultured in Eagle’s Minimum Essential Medium (ATCC 30-2003) with 10$\%$ fetal bovine serum supplement (Gibco) using Nunclon Delta dish (60 mm$\times$15 mm). Cells were passaged every 5–7 days at $\sim$80$\%$ confluence. SYTO 62 red nucleic acid stain was purchased from Invitrogen and used as directed. The culture medium of cells which had $\sim$80$\%$ confluence were removed and the cells were further rinsed by 1 mL DPBS (Dulbecco’s phosphate-buffered saline without $\hbox {Mg}^{2+}$ and $\hbox {Ca}^{2+}$). 1 $\mu$L of the 5 mM SYTO 62 stock solution was added to 1 mL of DPBS (without $\hbox {Mg}^{2+}$ and $\hbox {Ca}^{2+}$) to make a 5 $\mu \hbox {M}$ staining solution. The cells were stained in the culturing dish by adding 1 mL of the staining solution. The cells were incubated for 20 minutes, followed by 1 mL DPBS (without $\hbox {Mg}^{2+}$ and $\hbox {Ca}^{2+}$) rinsing. The cells were then dissociated from the dish surface by adding 1 mL trypsin. After 10 minutes of incubation, the cells were transferred to solution phase for centrifuging for 5 minutes at 1000 rpm. The supernatant was removed and 2 mL DPBS (without $\hbox {Mg}^{2+}$ and $\hbox {Ca}^{2+}$) was added to remove the unbonded dye molecule residuals. This cleaning process was repeated twice. The stained HEP G2 cells were redissolved back to 2 mL DPBS (without $\hbox {Mg}^{2+}$ and $\hbox {Ca}^{2+}$) for multicolor multifocal experiments.

### Image processing

All processing is done using self-written code in MALAB. Images from both color channels are cropped, normalized, and smoothed using a Gaussian filter. For processing of the images of mammalian cells in the 671 nm channel, image deconvolution is performed using the *deconvlucy()* function with the measured PSF for this color channel acting as an input to this process. After deconvolution, The 9 axial planes are interpolated to 27 planes. Next, the transparency values for each pixel are scaled exponentially based on the intensity value of each 3D voxel. The purpose of this step is to make the low intensity voxels more transparent to allow visualization of the brighter intensity voxels spanning the extent of the cells. These transparency values are then thresholded empirically and input into an open source function *vold3D()*^19^ which creates surface plots in all 3 dimensions resulting in the 3D volume of the cells. For the images of bacteria, deconvolution was first performed. Then, for every image in the acquisition sequence, end points of each bacteria were manually annotated in x,y and z. Once these are acquired, cylinders were plotted using the function *cylinderModel()* in MATLAB from these locations having a diameter of 500 nm.

## Supplementary Information

Supplementary Information 1.

Supplementary Information 2.

Supplementary Information 3.

Supplementary Information 4.

Supplementary Information 5.

Supplementary Information 6.

Supplementary Information 7.

Supplementary Information 8.

Supplementary Information 9.

## Data Availability

The data used to support the findings of this study are available from the corresponding author upon request.
