# Supplementary material for: Multicolor multifocal 3D microscopy using in-situ optimization of a spatial light modulator
Source: Sci Rep. 2022 Sep 29;12:16343. doi: 10.1038/s41598-022-20664-z (PMC9522655; doi:10.1038/s41598-022-20664-z)
Supplement: Supplementary file 1 — Supplementary Information 1. [file 41598_2022_20664_MOESM1_ESM.pdf]

Supplementary Information

Multicolor multifocal 3D microscopy using *in-situ* optimization of a spatial light modulator

M. Junaid Amin[1,2,3], Tian Zhao[1], Haw Yang[1], * and Joshua W. Shaevitz[2,3], *
[1]Department of Chemistry, Princeton University, Princeton, New Jersey 08544, USA
[2]Department of Physics, Princeton University, Princeton, New Jersey 08544, USA
[3]Lewis-Sigler Institute for Integrative Genomics, Princeton University, Princeton, New Jersey 08544, USA
*Corresponding authors: hawyang@princeton.edu, shaevitz@princeton.edu

Axial Point Spread Function (PSF)

To measure the PSF of the multifocal microscope, 200 nm fluorescent beads are immobilized on a coverslip and moved over a distance of 14 µm in the axial direction using the z-piezo stage in steps of 0.1 µm. XZ slices of the recorded images of a single bead in the acquired z-stack are shown for all the 9 subimages in Supplementary Fig. 1. Note how the in-focus position of the bead varies across the subimages. This data is acquired using an emission center wavelength of 671 nm and Δz = 900 nm.

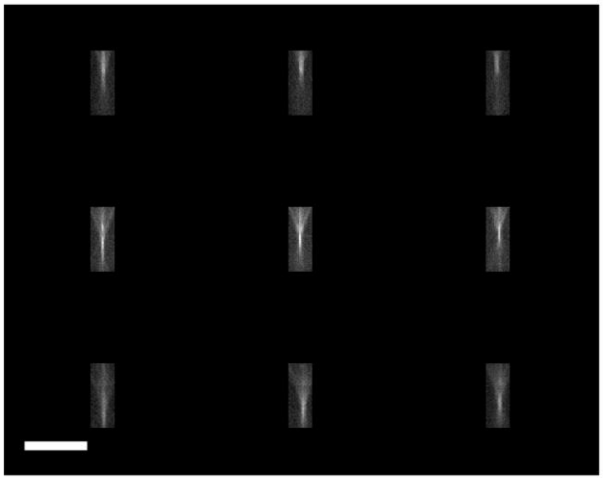

Supplementary Fig. 1. XZ slices of the 9 subimages showing the PSF of the multifocal microscope. For each subimage, the horizontal lateral extent of the XZ slice spans 2.6 µm while the vertical axial range is 14 µm. The emission of the fluorescence beads is 671 nm. The length of the scale bar is 5 µm.

Gaussian fitting of the PSF

The lateral and axial point spread functions of the 200 nm beads with 671 nm emission from 9 subimages are fitted by a Gaussian fitting routine. The fit results are shown in Supplementary Fig. 2. Note that the lateral FWHM values in Supplementary Fig. 2 vary across the subimages due to the different PSF shapes induced primarily due to the chromatic dispersion from the multifocal grating.

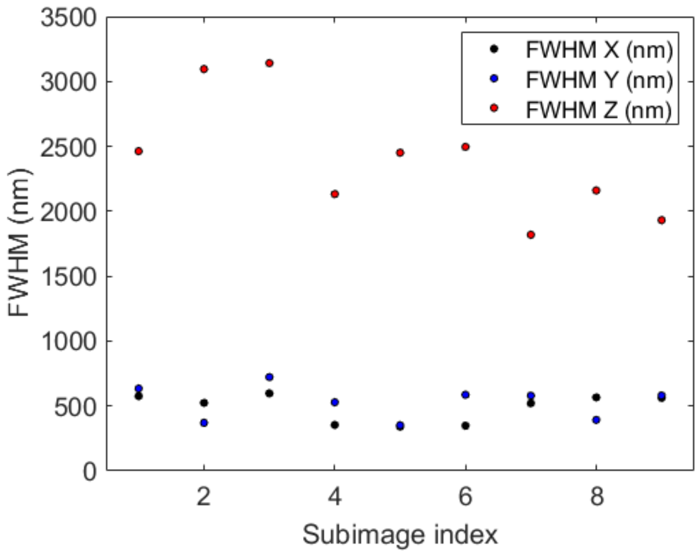

Supplementary Fig. 2. Results from Gaussian fitting of the PSF across all the 9 subimages.
